# Supplementary material for: Coix Seed Consumption Affects the Gut Microbiota and the Peripheral Lymphocyte Subset Profiles of Healthy Male Adults
Source: Nutrients. 2021 Nov 15;13(11):4079. doi: 10.3390/nu13114079 (PMC8618347; doi:10.3390/nu13114079)
Supplement: Supplementary file 1 [file nutrients-13-04079-s001.zip › Supplementary Figure S1.pdf]

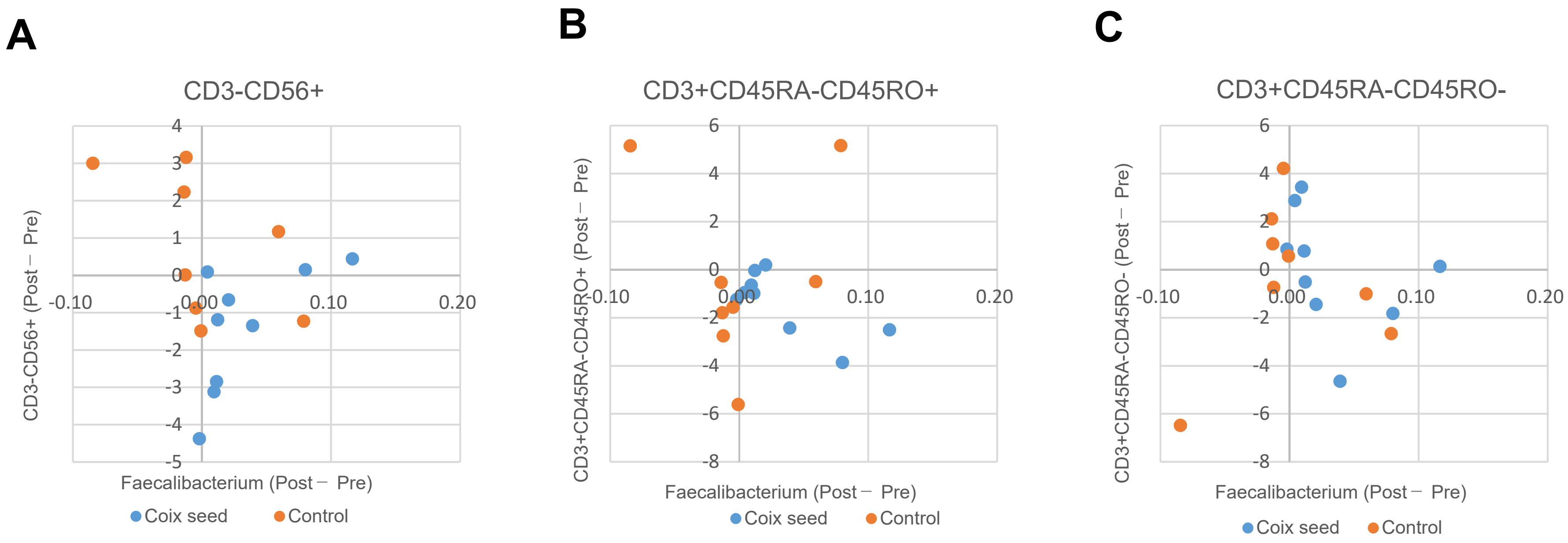

Supplementary Figure S1: Correlation of the changes in gut *Faecalibacterium* abundance with the changes in peripheral lymphocyte subsets (namely, A: $CD3^{-}CD56^{+}$; B: $CD3^{+}CD45RA^{-}CD45RO^{+}$; C: $CD3^{+}CD45RA^{-}CD45RO^{-}$) in the participants of the CS and the CN group, before and after the intervention described in the study
